# Evaluation of a universal 3-4 month Healthy Child Programme visit in a local area of England: a mixed methods study

**DOI:** 10.1016/j.puhip.2026.100837

**Published:** 2026-07-31

**Authors:** Nathan Davies, Denise Kendrick, Jessica Eve Jackson, Elizabeth Orton, Miriam Avery, Jo Leonardi-Bee, Hayden Bird, Holly Knight

**Affiliations:** a Nottingham Centre for Public Health and Epidemiology, School of Medicine, University of Nottingham, Nottingham City Hospital, Clinical Sciences Building, Hucknall Road, Nottingham, NG5 1PB, UK; b Centre for Academic Primary Care, School of Medicine, University of Nottingham, UK; c School of Health Sciences, University of Nottingham, UK; d Nottingham Centre for Public Health and Epidemiology, School of Medicine, University of Nottingham, UK; e University Hospital Southampton NHS Foundation Trust, Southampton, UK; f Centre for Evidence Based Healthcare, School of Medicine, University of Nottingham, UK; g Lincoln Institute for Rural and Coastal Health, University of Lincoln, UK

**Keywords:** Health visiting, Early intervention, Child development, Public health, Evaluation

## Abstract

**Objectives:**

The interval between the 6–8 week and 9–12 month mandated reviews in the Healthy Child Programme is lengthy and may lead to delayed intervention. In Rotherham, northern England, an additional universal 3–4 month review was introduced to support child development and earlier identification of parent and child needs. We aimed to evaluate its reach, acceptability, and association with developmental outcomes.

**Study design:**

Mixed methods study using administrative records, qualitative interviews and focus groups with parents (n = 31), nursery nurses (n = 7), and commissioners (n = 4).

**Methods:**

The association between uptake and recording of maternal mood by deprivation, ethnicity, maternal age, and parity for babies aged 3-4 months between January 2024 andFebruary 2025 was analysed using mixed-effects logistic regression. Developmental outcomes were compared (Ages and Stages Questionnaire (ASQ-3) scores at 9–12 months) for children eligible for the universal review versus pre-intervention cohort. Qualitative data were analysed using the Consolidated Framework for Implementation Research.

**Results:**

Uptake was approximately 80%, around 15% points lower than mandated reviews. Uptake was equitable across deprivation and ethnicity but lower among multiparous mothers (adjusted odds ratio (AOR) 0.29, 95% CI 0.22–0.37). Maternal mood recording was lower for mothers <20 years and for multiparous mothers (AOR 0.78, 95% CI 0.64–0.94). At 9-12 months, eligible children had higher odds of scoring above the close monitoring cut-off for problem solving (AOR 1.41, 95% CI 1.03–1.93), with no significant differences in other ASQ-3 domains. Parents valued reassurance, tailored developmental advice, and local signposting, though some noted inconsistent advice, maternal mood assessment and unclear visit purpose. Staff endorsed the visit's developmental focus but reported capacity pressures.

**Conclusions:**

The universal 3–4 month review was broadly acceptable, and associated with higher ASQ-3 problem-solving scores. Communicating visit purpose and consistent mood assessment is required.

**What this study adds**

• Maintaining equitable access is achievable, but lower uptake was reported compared to mandated visits.
• Early developmental gains in child problem-solving were observed, but inconsistent maternal mood screening, particularly among younger mothers, highlights the importance of strengthening routine wellbeing assessment and standardised data collection for evaluation and commissioning.

**Implications for practice and policy**

• An additional universal contact at 3-4 months may help address the long gap between mandated Healthy Child Programme reviews, offering parents timely developmental reassurance, tailored advice, and improved signposting to wider support services.
• Reduced engagement among multiparous families and parental reporting of uncertainty of visit purpose imply that defining clear purpose of visit and communication with parents may support higher, more equitable uptake.

## 1 Introduction

In England, the Healthy Child Programme (HCP) is a national framework for promoting health in the perinatal period to age 19 [1]. The programme is commissioned by local authority public health teams and usually delivered by NHS providers through Child and Family Health teams. These teams can include public health nurses (health visitors), children's nurses and early years practitioners (nursery nurses). The HCP involves comprehensive early needs assessments for tailored interventions and acts as a key pathway for accessing wider specialist services [[2], [3], [4]].

The HCP includes five mandated core contacts (antenatal, new birth, 6-8 weeks, 9-12 months, and 2-2.5 years) that monitor growth, immunisations, development and parental wellbeing [1]. A universal 3-4 month review had historically been established as part of the service schedule, but was not mandated in the HCP [5]; the latest national HCP guidance suggests that completing additional *targeted* reviews at 3 months may be particularly valuable to support feeding advice and delaying weaning and addressing parental coping and resilience [6]. In a recent qualitative study, HCP staff described the loss of this universal contact as a missed opportunity for timely weaning advice, early weight management and overall assessment [7]. Findings from a rapid evidence review suggests the gap between the 6-8 week and 9-12 month reviews may be too long for many families [5].

The general quality of evidence for timing of postnatal visits is generally low, with the National Institute for Health and Care Excellence finding little evidence of a sufficient standard to inform their guideline on postnatal care [8]. The limited evidence does indicate positive outcomes for both children and families. A pilot 3-4 month visit recorded 100% take-up and reported better parent-practitioner relationships, improved staff morale and detection of issues for which early help could be provided [9].

Equity is an important consideration for any new addition to the HCP schedule. Recent national analyses of administrative data for England shows that, while early mandated reviews typically achieve high and broadly equitable coverage across area-level deprivation [10], there is evidence that non-White children may receive less support from the HCP in the first year of life [11] and that babies whose mothers were of Bangladeshi, Pakistani or Black origin had lower coverage at the 6-8 week check than those of White British ethnicity [12].

In Rotherham, a relatively deprived town in northern England, local authority commissioners used national government grant funding to introduce a 3-4 month home visit as part of an integrated early years offer linking the HCP with broader local support for families, which was previously offered only to families needing extra support. The local area piloted broader eligibility in September 2023 for first-time and higher-risk parents and rolled out the visit universally in January 2024. Delivered by nursery nurses, the visit included Ages and Stages Questionnaire 3 (ASQ-3) developmental review, advice on feeding, safety and weaning, and review of parental wellbeing.

Given that no studies since 2015 have directly evaluated the addition of a universal 3–4 month visit, our evaluation sought to understand the demographic reach of the new 3-4 month universal visit, the association of the visit with developmental outcomes, and the acceptability of the visits to parents/primary carers (hereafter referred to as ‘parents’) and staff.

## 2 Methods

### 2.1 Study design

A mixed methods process evaluation was undertaken to assess the reach, acceptability, and association with developmental outcomes of the universal 3-4 month home visit review. A study protocol was published on Open Science Framework (OSF) before data collection commenced [13]. We took a convergent parallel approach in which qualitative and quantitative data collection and analysis were conducted concurrently and independently, before findings were integrated and interpreted together [14]. Quantitative analysis of routinely collected administrative data was complemented by qualitative interviews and focus groups with parents, practitioners and local stakeholders. Administrative HCP data were extracted for all those eligible for an HCP visit in the local area between October 2019 and February 2025. The evaluation design was informed by the Consolidated Framework for Implementation Research (CFIR) to systematically explore implementation processes [15].

### 2.2 Quantitative analysis: reach

To assess reach, we analysed the proportion of eligible infants who received a 3–4 month visit, whether the visit was completed within the recommended time window, whether maternal mood was recorded, and trends in uptake over time. R version 4.4.1 was used for analysis [16].

Equity of access was assessed by comparing rates of completion, on-time completion, and recording of maternal mood across deprivation quintiles (with quintile 1, the most deprived, as the reference group), ethnic groups (ONS 5-group classification, White as the reference group), maternal age categories (<20 [reference group], 20–24, 25–29, 30–34 and ≥ 35 years), and gravida (primigravida and multigravida). We used mixed-effects logistic regression to adjust for these factors as well as month of birth and included the three NHS team areas as a random intercept to account for nesting at team level. Results are reported as adjusted odds ratios (AOR) with 95% confidence intervals (CI). Where covariate data were missing, we used multiple imputation with imputation models specified for each variable type (logistic or multinomial logistic) to reduce the risk of bias and retain the full analytic sample [17].

### 2.3 Quantitative analysis: association with child development

To assess association with child development, we analysed child development outcomes at the 9-12 month ASQ-3 review, a validated parent-completed developmental screening tool with established age-specific thresholds [18]. Each ASQ-3 domain was categorised as “below cutoff”, “in the monitoring zone”, or “above the monitoring cutoff” using ASQ-3 validated thresholds [18]. For reporting purposes, these were dichotomised into “above” versus “near” or “below” the monitoring cutoff. Those above the monitoring cutoff are considered to have expected levels of development. As a population-level intervention, we used multiple logistic regression to compare expected levels of development for children who were eligible for the 3-4 month visit (not just those who received the visit) versus those who were born before its introduction. Children were included if they were born between January 2022 and March 2024 (excluding those born pre-2022 in periods with greatest COVID-19 restrictions) and had a completed 9-12 month ASQ-3 record. The intervention cohort comprised those born from September 2023 onwards (encompassing those eligible for universal rollout) and the pre-intervention cohort those born before June 2023. Children born between June and August 2023 were excluded as the majority of this cohort had their visit during the non-universal 3-4 month visit pilot phase. The analyses adjusted for deprivation quintile, ethnic group, maternal age category and gravida (categorised as in reach section). Area was evaluated as a random intercept but was removed because its inclusion produced non-convergence, suggesting the between-area variance was too small for reliable estimates. We used multiple imputation for covariates as described in the Reach section [17].

### 2.4 Qualitative data collection

We used semi-structured interviews and focus groups to explore parent and professional experiences of the visit. Parents eligible for the visit between December 2024 and March 2025 (around 800) were invited by text or HCP app notification to interview. Parents were eligible to take part if they were the primary carer of a child eligible for the universal visit in the preceding three months, were aged 16 or over, could give informed consent, and could understand conversational English (unless a translator was present); those whose child received enhanced support through the 0–19 service were excluded. Staff participants comprised all nursery nurses delivering the visit and local authority staff involved in its development, oversight, or commissioning. Two parent focus groups (one group advertised through Facebook and one taking place with usual participants of a breastfeeding group at a local mosque) and two staff focus groups (one for nursery nurses delivering the visit and one for local authority commissioners) were held. Parents received a £20 shopping voucher; staff did not. Interview and focus group schedules were mapped to CFIR domains (Supplementary Material A). All sessions were audio-recorded and transcribed verbatim. We intended to purposively sample by parent age, ethnicity, postcode IMD, and gender; however, response rates were low, and the final sample was one of convenience.

### 2.5 Qualitative data analysis

Data were coded thematically using Taguette [19] and MS Excel. HK and MA independently reviewed the parent interview and focus group data and coded for emerging themes using relevant CFIR domains and constructs [15]. A construct is a factor within the CFIR framework that helps analyse how, why and where an intervention is implemented, and how successful it has been; a domain is an overarching category that contains several constructs. HB followed the same process for the staff interviews and focus groups. A ‘critical friend’ approach [20] was taken to cross-check the findings, which included HK, MA and HB reviewing coding for both parent and staff data and triangulating themes from both sources. The concept of information power, which accounts for study aims, quality of interview dialogue, complexity of theory and specificity of sample strategy, was used to assess the adequacy of data collected to draw conclusions [21].

### 2.6 Deviation from protocol

We originally intended to analyse onward referrals to other services, immunisation uptake and the prevalence of breastfeeding as outcome variables. However, onward referrals were documented only within multi-purpose free-text fields, which limited their extractability and consistency. Immunisation data were not stored within the same HCP dataset and were therefore unavailable for analysis. In addition, too few breastfeeding observations were recorded in the pre-intervention period to permit a robust assessment of the visit's effect on breastfeeding.

The research team also planned to assess parental knowledge and confidence using a pre- and post-visit survey facilitated by the local authority for those attending the 3–4 month review. This survey was not completed in time for inclusion in the current evaluation. Nonetheless, qualitative data allowed rich exploration of themes relating to parental confidence and knowledge following the visit.

### 2.7 Public contribution

This evaluation was co-designed by the PHIRST Light team with the local authority, the local charity sector, and public contributors through a series of online and in-person workshops. The focus of these workshops was to gain consensus on the primary research questions and explore the most appropriate methods to answer these. To ensure ongoing involvement, we formed a project-specific patient and public involvement group of local parents, recruited through an existing parent voices group and snowball sampling, who worked alongside the team's public advisory group. In addition to designing recruitment materials and advising on interview schedules, public contributors co-produced recruitment strategies, helped identify suitable community settings, and supported the set-up and running of the parent focus groups.

## 3 Results

### 3.1 Quantitative findings: reach

**Fig. 1:**
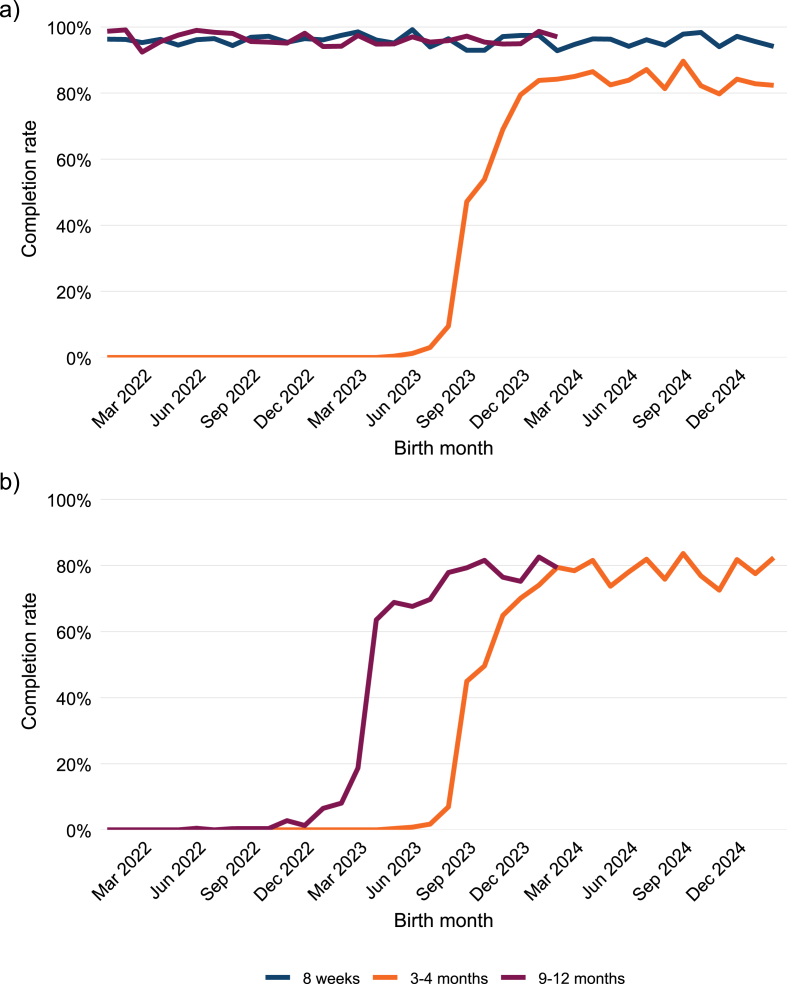
a) HCP visit completion by visit period and birth month b) ASQ-3 completion by visit period and birth month (completed visits only).

By January 2024, universal 3-4 month visit rates stabilised at around 80%, below the 95% visit rates of the 6-8 week and 9-12 month visits (Fig. 1a). On average, just under 200 babies and their families received a 3-4 month visit each month. Recording of the ASQ-3 at the 3-4 month visit began for babies born in June 2023. For babies who received a visit, the rate of completion of the ASQ-3 stabilised at around 80% in January 2024, which is comparable to the ASQ-3 completion rate for the 9-12 month review (Fig. 1b).

Overall, uptake of the 3–4 month visit appeared equitable across levels of socio-economic deprivation, with no clear trend from most to least deprived areas. No differences were found for ethnicity or maternal age groups; however, families with more than one child had around 70% lower odds of receiving a visit (AOR 0.29, 95% CI 0.22 - 0.37). (Table 1).

**Table 1:** Adjusted odds ratios for completed 3-4 month visits by child and mother demographics.

Demographic	N	Adjusted odds ratio (95% CI)	p-value
IMD (global)			0.26
IMD 1 (most deprived)	1475	**Ref**	
IMD 2	770	0.758 (0.573 – 1.004)	0.05
IMD 3	410	0.987 (0.702 – 1.387)	0.94
IMD 4	458	1.053 (0.741 – 1.494)	0.78
IMD 5 (least deprived)	154	0.97 (0.587 – 1.602)	0.91
Ethnicity (child) (global)			0.34
White	2517	**Ref**	
Asian	449	0.766 (0.530 – 1.109)	0.16
Black	217	1.307 (0.785 – 2.176)	0.30
Mixed	171	0.87 (0.537 – 1.410)	0.57
Other	42	0.618 (0.246 – 1.550)	0.31
Age of mother (AoM) (global)			0.46
AoM <20 years	107	**Ref**	
AoM 20-24 years	512	1.055 (0.501 – 2.218)	0.89
AoM 25-29 years	999	1.331 (0.642 – 2.758)	0.44
AoM 30-34 years	1089	1.223 (0.590 – 2.535)	0.59
AoM >34 years	660	1.037 (0.494 – 2.179)	0.92
Gravida			
Primigravida	1258	**Ref**	
Multigravida	1539	0.287 (0.224 – 0.368)	<0.001

Mixed-effects logistic regression adjusted for IMD quintile, age of mother, gravida, ethnicity, birth month, NHS area (random intercept). Birth month excluded from table for clarity. Missing covariates imputed for AOR calculation.

Table 2 presents maternal mood recording at visits. Maternal mood recording was consistent across deprivation quintiles and ethnic groups. However, mothers older than 20 years had at least two-and-a-half the odds of mood recording compared to those under 20 years. Women who had been pregnant before had lower odds of having their maternal mood recorded compared to those for whom it had been their first pregnancy (AOR 0.78, 95% CI 0.64–0.94).

**Table 2:** Adjusted odds ratios for maternal mood recording at completed 3-4 month visits by child and mother demographics.

Demographic	N	Adjusted odds ratio (95% CI)	p-value
IMD (global)			0.910
IMD 1 (most deprived)	1220	**Ref**	
IMD 2	611	0.951 (0.758 – 1.193)	0.663
IMD 3	314	1.033 (0.770 – 1.385)	0.829
IMD 4	368	1.035 (0.798 – 1.341)	0.796
IMD 5 (least deprived)	119	0.869 (0.583 – 1.295)	0.490
Ethnicity (child) (global)			0.568
White	2031	**Ref**	
Asian	371	0.918 (0.713 – 1.183)	0.510
Black	177	1.105 (0.790 – 1.546)	0.558
Mixed	137	1.242 (0.855 – 1.805)	0.256
Other	33	0.745 (0.347 – 1.601)	0.451
Age of mother (AoM) (global)			<0.001
AoM <20	96	**Ref**	
AoM 20-24	436	2.791 (1.592 – 4.891)	<0.001
AoM 25-29	854	3.451 (1.995 – 5.968)	<0.001
AoM 30-34	878	3.380 (1.943 – 5.882)	<0.001
AoM >34	520	3.084 (1.743 – 5.456)	<0.001
Gravida			
Primigravida	1129	**Ref**	
Multigravida	1161	0.775 (0.636 – 0.944)	0.012

Mixed-effects logistic regression adjusted for IMD quintile, age of mother, gravida, ethnicity, birth month, NHS area (random intercept). Birth month excluded from table for clarity. Missing covariates imputed for AOR calculation.

### 3.2 Quantitative findings: ASQ-3 scores

We compared the 9-12 month ASQ-3 scores for those who were eligible for the 3-4 month visit (post-intervention) against those who were not (pre-intervention). The two cohorts were similar in demographic profile. Before imputation, missingness among the adjusted covariates was, for pre- and post-cohorts respectively, 2.6% and 2.9% for maternal age, 1.3% and 2.4% for ethnicity, 6.3% and 10% for deprivation and 19.5% and 21.3% for gravida (Supplementary Material B). At the 9-12 month visit, children eligible for a universal 3-4 month visit had higher odds of scoring above the close monitoring cutoff in the ASQ-3 domain of problem solving (AOR 1.41, 95% CI 1.03-1.93) than the pre-intervention cohort, indicating better problem-solving development. There were no significant differences in communication, gross motor, fine motor and personal social domains when compared to the pre-intervention cohort (Table 3). An analysis of mean difference in scores is presented in Supplementary Material C.

**Table 3:** Comparison of ASQ-3 scores above the close monitoring zone cutoff at 9-12 month visit, pre-vs post-intervention.

ASQ-3 domain	AOR (95% CI)	p-value
Communication	1.119 (0.818 – 1.530)	0.31
Gross motor	1.188 (0.901 – 1.567)	0.16
Fine motor	0.902 (0.663 – 1.227)	0.58
Problem solving	1.411 (1.033 – 1.927)	0.03
Personal social	1.054 (0.762 – 1.458)	0.79

Multiple logistic regression adjusted for IMD quintile, age of mother, gravida, ethnicity, birth month. Missing covariates imputed for AOR calculation.

### 3.3 Qualitative findings

Of the 197 parents who expressed interest in participating by responding to an initial text, a convenience sample of 15 attended a semi-structured interview, all of whom had taken up the visit. 182 parents did not respond to requests to arrange an interview, meaning intended purposive sampling could not take place. Focus group 1 (FG1) included parents who responded to a Facebook group advert (both those who had taken up and opted out from the visit (n = 7)) and focus group 2 (FG2) took place at a local mosque with a Pakistani and Urdu-speaking community breastfeeding group (n = 9) who agreed to take part on the day of data collection. Further details, including demographic breakdown, are available in Supplementary Material D.

All 24 nursery nurses involved in delivering the 3-4 month visit were invited to take part in a focus group; n = 7 participated. Four local authority stakeholders selected for greatest involvement in the intervention participated in a mixture of focus groups and individual interviews. A synopsis of results is presented in Table 4 by the five CFIR domains and constructs, with representative quotes. A full table is presented in Supplementary Material D.

**Table 4:** Synopsis of qualitative findings by CFIR domain and construct.

CFIR domain	Construct	Summary of key factors	Illustrative quote
**Innovation**	**Relative advantage**	Parents valued an additional contact between mandated reviews; staff felt the visit enabled earlier identification of developmental issues and offer of earlier support.	“It is a great support … with my first I didn't get that (visit) and … you're in the dark”. (Parent 009) “So you potentially are looking at more referrals if they've not had this contact as a health professional”. (Nursery nurse)
	**Design**	Home visits felt convenient and promoted openness. Longer appointments allowed fuller discussion. Parents reported some variability in content and advice, such as mixed advice on weaning. Some parents felt the visit should have been at 4-5 month to better align with weaning; staff believed 3-4 months was better timed to counter misinformation and identify issues early.	“It lasted longer. It was more informative compared to the eight week review”. (Parent 005) “It's like a crucial time to speak with parents because that information can be reiterated, what's not always taken in from the new births or the six to eight weeks." (Nursery nurse)
	**Complexity**	Parents largely viewed the visit as low burden. Staff noted it was their favoured visit but reported it added pressure on workload and capacity.	“Just by reading the form I kind of understood what to do”. (Parent 004) “We like to do that visit, but it has put extra pressure on capacity”. (Nursery nurse)
	**Adaptability**	Advice was generally perceived as culturally appropriate. Parents valued tailored guidance on developmental needs.	“And I were really a bit shocked thinking ‘great she gave me the advice for circumcision and that’.” (Parent FG2)
**Individuals**	**Capability**	Visit improved parental knowledge and confidence in milestones. The ASQ-3 helped understanding but could generate worry; some experienced parents found it less relevant.	“Very positive and very informative. I gained a lot of confidence from the review in terms of how to care for my baby, next steps with weaning and teething”. (Parent 005) “With my daughter it were good, I needed the support, but with each time now … so my daughter's only just turned two so it's still quite fresh having a baby, do you know what I mean, it's not like I don't know what I'm doing”. (Parent 003)
	**Need**	Some parents felt mother's wellbeing was covered in detail, others felt it was insufficiently screened. Some staff recognised a need for further training in mother's mental health.	“I think it really can't be underestimated, the importance around the mental health discussion for parents. I think for me it felt like sometimes that was a little bit glossed over or not really given the opportunity to talk about that enough”. (Parent 014)
	**Innovation deliverers**	Positive relationships with nursery nurses and perceived professionalism were central to parent trust. Occasionally judgemental communication undermined rapport.	“I definitely felt heard. She answered all my questions, gave me references if I needed to follow anything up with mental health or with weaning or anything. She definitely listened. Didn't feel rushed at all. She were really lovely”. (Parent 006) “She'd judge you without … um … like you could never have done right in her eyes and you've got to follow the book's way in being a mum, being a parent, a baby should do this at this time". (Parent 003)
**Inner setting**	**Communication**	Parents wanted clearer information about the purpose and length of the visit. There were differing understandings of whether the visit was optional or mandatory. Parents valued signposting to local services.	“The text that we got sent with the date just said 3-4 month review, didn't give us any information as to why or include a link or anything which might have been helpful”. (Parent 014) “She'd signposted to … baby massage stuff and the play groups and weaning courses”. (Parent 012)
	**Work & IT infrastructure**	Parents found scheduling and text communication helpful. Staff continuity across visits was valued but difficult to achieve because different professionals conducted different visits.	“I think I just didn't see the same person enough. And I think because the visits are very few and far between, I wouldn't say that I built very good rapport". (Parent FG2)
**Outer setting**	**External pressures**	National uncertainty around Family Hub funding affected local commissioner confidence in being able to sustain the visit.	“It could be that they will extend the programme to be effectively across the whole country, which might then mean that we get a smaller slice of the cake.” (Local authority stakeholder)
**Process**	**Reflecting and evaluating**	Staff highlighted the importance of ongoing data collection to evidence impact and inform commissioning decisions. Opportunity cost for funding other child public health interventions was a key concern.	“I think as long as we can evidence that, you know, it's having an impact locally, then I think it's kind of really I guess for myself, and (staff member) to make the financial case and I think that's always a difficult one … is that three to four month review more important than anything else we do.” (Local authority stakeholder)

## 4 Discussion

### 4.1 Summary of findings

Our evaluation found that parents largely valued the re-introduced universal 3–4 month review, although were not always clear on its purpose. Staff highlighted both its perceived benefits and the pressures of delivering an additional universal review within a stretched service. Uptake stabilised at around 80 percent and was broadly equitable by deprivation, ethnicity and maternal age, though younger mothers were less likely to have maternal mood recorded. Children receiving the universal contact had higher problem-solving scores at 9–12 months than those in the pre-intervention cohort, with no clear differences in other ASQ-3 domains. Integrated working between health and local authority practitioners appeared to support the visit's wider signposting function, but gaps in data collection remained.

### 4.2 Comparison with literature

Staff reported pressures associated with incorporating a new universal offer within an already stretched 0–19 service. In a recent UK study health visitors described the “stretching” being due to the service as having to “fill the gaps” in both health and children's services [22] - a possible consequence of it being a service that straddles across health and local authority provision. However, the visit was described as offering meaningful opportunities for support and signposting to wider services, a function which is likely enhanced by the integrated nature of the service.

Quantitative evidence indicates broad equity in uptake by area-level deprivation, maternal age and ethnicity. Although early mandated HCP visits in the UK demonstrate high and relatively even uptake across deprivation gradients [10], the finding of equitable uptake by ethnicity is particularly important, as ethnic equity is not consistently reported within HCP delivery in the UK [11,12]. This was also in the context of overall uptake stabilising at around 80 percent, lower than the 6-8 week and 9-12 month mandated visits; one might expect greater variation in population reach across groups when a service is non-mandated and uptake is lower. Based on our qualitative analysis, the lower uptake likely reflected limited parental clarity about purpose (an underlying issue for even mandated reviews [23,24]). Uptake was notably lower among multiparous mothers; in our interviews and focus groups, experienced parents felt less need for early contact.

Children in the post-intervention cohort recorded significantly higher ASQ-3 problem-solving scores at 9-12 months compared with those seen before the universal contact was introduced. The problem-solving domain has moderate sensitivity to predict future cognitive delay [25]. Qualitative accounts indicate that personalised guidance from nursery nurses to parents on developmental needs during the visit and referrals to community settings may form part of the explanation for this difference. This is consistent with evidence that responsive feedback and enriched sensory environments facilitate early cognitive development [26]. However, whether a difference confined to a single screening domain is clinically meaningful is uncertain. All five ASQ-3 domains were pre-specified and thus reported equally without correction for multiple comparisons; accordingly, an isolated significant result may be a chance finding, although the sensitivity analysis in Supplementary Material C is consistent with a genuine association. The development analysis was restricted to children with a completed 9-12 month ASQ-3, and both visit uptake and ASQ completion were incomplete (around 80%), and multiparous mothers were less likely to receive the visit. If those completing the review differed systematically between cohorts, residual confounding may remain despite adjustment and the observed problem-solving association may be over- or underestimated. In addition, there may be residual confounding from unmeasured confounders such as secular trend or increased provision of other forms of family support in the local area. No statistically significant differences were observed in other ASQ-3 domains, although this does not definitively rule out an effect of the visit in these areas of development. We did not capture component-level fidelity in the administrative data; qualitative accounts indicated some variability in delivery across staff.

Maternal mood recording was similar across deprivation and ethnic groups, but younger and multiparous mothers had lower odds of having their mood documented, despite higher risk of postpartum mental illness in younger mothers[27]. Both parents and staff regarded maternal wellbeing discussions as integral to the visit, yet both groups noted inconsistent implementation and variable recording practices, which follow-up conversations indicated may be related to the existence of multiple methods and places to record the mother's mental health. A clearer, standardised approach to documentation and greater attention to how mood is discussed with younger and multiparous mothers may support both equity and data quality. Further study of differences in maternal mood using validated measured for those receiving additional universal visits is warranted.

Our study has several strengths. To our knowledge, it is the first comprehensive evaluation of a universal 3-4 month HCP visit in England. We used a population-level administrative dataset for analysis, which included key covariates. By combining quantitative data with qualitative data structured through CFIR, a comprehensive implementation framework, we were able to contextualise quantitative findings and report findings in areas where quantitative data were not available, such as onward referrals and signposting. There was significant public input into designing the study.

The study also has limitations. It is a single-site study which limits generalisability to other contexts, although it was applied to the whole 3-4 month population. Length of follow-up was limited to the 9-12 month visit, and we were unable to consider quantitative data on immunisations, referral to other services or feeding practices. The lack of interoperable data systems across different NHS trusts and the local authority suggested that although co-location and joint working appeared to be taking place, there is room for improvement in data-sharing integration. Finally, our qualitative interview participants were sampled for convenience rather than purposively; although we supplemented this with two additional focus groups with participants who represented missing demographics, our sample lacked information power for those who did not take up the offer of a visit.

### 4.3 Conclusions

This mixed-methods evaluation shows that re-introducing a universal 3–4 month health-visiting contact is acceptable to families and deliverable within an integrated 0–19 service, albeit with additional pressure on staff. The visit offered valued developmental guidance and signposting and achieved broadly equitable reach, although there was lower overall uptake than mandated reviews, particularly amongst multiparous mothers. Higher problem-solving scores in the post-intervention cohort suggest an association with developmental benefits, though longer follow-up and studies designed for rigorous causal inference are required. Inconsistencies in maternal mood assessment, particularly for multiparous and younger mothers, indicate an area for improvement. As the first evaluation of this model in England for at least a decade, these findings can support other local areas considering implementation and guide multi-site research to assess longer-term outcomes.

## Ethical statement

Ethical approval was granted by the University of Nottingham Faculty of Medicine and Health Sciences Research Ethics Committee (reference FMHS 44-1224).

## Ethical declaration

The author is an Editorial Board Associate Editor for this journal and was not involved in the editorial review or the decision to publish this article.

## Funding

The study was funded by the National Institute for Health and Care Research (NIHR PHIRST Light, Award ID NIHR135190). The views expressed are those of the author(s) and not necessarily those of the NIHR or the Department of Health and Social Care.

## Declaration of competing interest

The authors declare that they have no known competing financial interests or personal relationships that could have appeared to influence the work reported in this paper.
