# Supplementary material for: Evaluation of a universal 3-4 month Healthy Child Programme visit in a local area of England: a mixed methods study
Source: Public Health Pract (Oxf). 2026 Jul 31;12:100837. doi: 10.1016/j.puhip.2026.100837 (PMC13473960; doi:10.1016/j.puhip.2026.100837)
Supplement: Supplementary file 1 [file mmc1.docx]

**Supplementary Material A: Interview and focus group schedules**

**Public Health Intervention Responsive Studies Teams (PHIRST):**

**PHIRST Light Evaluation of the Rotherham Healthy Child Programme.**

**2009 CFIR-Based Interview Schedule for Parents**

**Introduction (5 minutes)**

Welcome and brief explanation: *"* *Thank you for taking the time to speak with me today. We’d like to hear about your experience with the new 3–4-month check* for mums/dads/carers and babies. This check is part of a trial designed to offer more support during this important stage, including advice on your baby’s development, your well-being, and practical tips for parenting. Your feedback will help us understand what works well, what could be improved, and how we can make this service as helpful as possible for families like yours.* The interview will take up to an hour and your insights will only be used to help us improve the service for families. So today we aim to*:*

- **Hear About Your Experience** and what you think worked well and needs to improve.
- **See how helpful it was** in supporting your baby’s development, your well-being, and your family, and if it helped build your confidence.
- **Check how it fit into family life,** in terms of the timing, structure, and tools (like the asq-3) worked well with your routine and were easy to use.
- **Get ideas from parents** to make this extra check better and more helpful for families.

*Note the 3-4 month check is also know as: a review; check-in, appointment; service or visit – adapt to parent being interviewed

**(For interviewer: Questions in bold, with probes beneath)**

1. **Could you please tell me a bit about your experiences of health and care services as a parent?**

Probes:

- First child or more than one child
- Parent experience and experience of baby/ies?
- If more than one child is this your first experience with the service.
- History/background on health visits to date.

Domain: Innovation Characteristics

*Innovation Source*

2. **How and when did you find out about the 3–4-month review?**
   Probes:
   - Was it through your health visitor, GP, or someone else?
   - Did you feel the information about the review was clear?
   - Did anyone explain why this review was introduced?
3. **When you first heard about it, what did you think it was about?**
   Probes:
   - Did you know who you were going to have the review with e.g., Health Visitor, Midwife, Staff Nurse, Nursery Nurse
   - Did it seem like something that would be helpful for you and your baby?
   - Did you have any concerns about attending the review?

*Evidence Strength & Quality*

4. **What did you know about the goals of the review?**

*[Also addresses Knowledge & Beliefs about the Innovation]*
Probes:

- How did the health professional explain the goals of the review?
- Did they discuss topics like play, safety, or weaning during the session?
- Did you feel they explained the purpose in a way you could relate to?

5. **How useful and practical was the advice given by the health professional during this review?**
   Probes:
   - For example, around weaning or home safety.
   - Did you feel confident applying the advice at home?
   - Were there any pieces of advice that stood out as particularly useful or not helpful?

*Relative Advantage*

6. **How did this review compare with any other reviews you have had following the birth of your baby?**
   Probes:
   - Did it address any concerns or questions you’ve had since the earlier visit?
   - Was there anything new or unexpected you found helpful?
7. **Do you think the timing of this review was right for your family’s needs?**
   Probes:
   - Why/why not? What could have been different?
   - What do you think might have been missed without this review?

*Adaptability*

8. **How did the review fit into your family’s life?**
   Probes:
   - Did the timing of the appointment work for you?
   - Would more flexibility, like evening or weekend appointments, help?
9. **Was there anything about the way the review was delivered that could be improved?**
   Probes:
   - How easy was it to schedule? Or attend? Or to communicate with the health professional.
   - Were there any challenges to arranging the review? Other commitments, like work or childcare?
   - Would a different format (e.g., online or group session) have helped?

*Complexity*

10. **Was the review straightforward and easy to follow?**
    Probes:
    - Were the instructions or materials clear and simple to understand?
    - Did you feel rushed or confused at any point?
    - Did you feel heard during the conversation?
11. **How comfortable did you feel during the review?**
    Probes:
    - How openly did you feel you could discuss matters that worried you? did you feel heard?
    - How about the questionnaire, how easy was it to follow? Did you receive the questionnaire in advance or did the health professional go through the questions with you?
    - Did the health professional discuss the results with you in a clear way?
    - Would you have liked more guidance on how to use the questionnaire?

*Compatibility*

12. **Do you think the review discussed all the topics which are important to you parenting your baby at this age?**
    Probes:
    - Did the review cover areas you were most interested in, like feeding or safety?
    - Was there anything you wanted to discuss but didn’t get a chance to?
13. **How did you feel about the advice you were given?**
    Probes:
    - Did you feel the recommendations fit with your parenting style?
    - Were there any suggestions that didn’t feel relevant or helpful to you?

Domain: Individual Characteristics

*Knowledge & Beliefs about the Innovation*

14. **Has your experience with this review changed your expectations for health visits in the future?**
    Probes:
    - Do you now expect similar support in other health check-ups?
    - Has it made you feel more engaged with health services?

*Self-Efficacy*

15. **Did the review change your confidence to support your baby’s growth, manage illnesses, or prepare for milestones like weaning?**
    Probes:
    - Did the review leave you feeling prepared for the next stage in your baby’s development?
    - Were there specific tips or guidance that boosted your confidence?

Domain: Outer Setting

*Patient Needs & Resources*

16. **Did you feel you were given enough information about other resources available through the Family Hubs, like activities or support groups?**
    Probes:
    - Were you encouraged to attend any follow-up sessions or groups?
    - Did the resources seem relevant to your needs?

*Follow-Up Ref/errals*

17. **Has this review led to any follow-up contacts or additional services to support your baby’s health or your own health?**
    Probes:
    - Were the referrals easy to arrange and access?
    - Did they address specific concerns effectively?

**Closing Questions (5–10 minutes)**

- **Overall, how would you describe your experience with the review?**
  Probes:
  - What stood out most to you about the experience?
  - Is there anything you would change?
- **Do you feel that the service should continue offering the 3-4 month review to all parents?**

Probes:

- Why, why not
- **Would you recommend it to other parents? Why or why not?**
  Probes:
  - What would you tell other parents about this review?
  - Are there reasons why someone might not find it helpful?
- **Is there anything else you’d like to share about your experience or ideas for improvement?**
  Probes:
  - Are there any final thoughts or suggestions you’d like to add?
  - Do you think we missed anything important in our questions?

**Wrap-Up (2–3 minutes)**

**Thank the parent for their time and insights:**
*"We truly appreciate you sharing your experience. This will help us improve the service to better support families."*

- *Ask how best to get £20 gift voucher to carer.*
- *Would you like us to keep you updated on the programme’s development?*

**Public Health Intervention Responsive Studies Teams (PHIRST):**

**PHIRST Light Evaluation of the Rotherham Healthy Child Programme.**

**CFIR-Based Focus Group Schedule for Parents**

1. **Could you please tell me a bit about your experiences of health and care services as a parent?**

*Probe: First child or more than one child; If more than one child is this your first experience with the service, history/background on health visits to date.*

Domain: Innovation Characteristics

*Innovation Source*

2. **Can you please tell me about what you think the 3-4 month health visit or check-in involves?**

*Probe: If you heard about the 3-4 month visit before today how did you find out about the visit?*

*Evidence Strength & Quality*

3. **If your child had the 3-4 month visit can you share your own experience with the group of what happened please?**

*Probe: what advice was given by the health professional during this review and how it was delivered*

*Probe: How useful was the advice provided by the person conducting the visit?*

*Probe: knowledge, communication approach, openness to ask questions…*

*Relative Advantage*

4. **For everyone – what are your thoughts on the timing of the visit at 3-4 month?**

*Adaptability*

5. **For everyone - Are there any suggestions you would like to make about how health visits could be improved?**

*Probe: 3-4 month visit specifically – if so how, skills of staff, place and time, signposting to services; scheduling; in person/virtual/groups one to one*

*Complexity*

6. **The Healthy Child Programme seeks to support babies’ development by using a questionnaire called the Ages and Stages questionnaire, if you have experience of the questionnaire could you share these please?**

*Compatibility*

7. **Based on what you know does the 3-4 month visit focus on the issues that matter to you and you child/ren?**

Domain: Individual Characteristics

*Knowledge & Beliefs about the Innovation*

8. **Can you describe if you think the 3-4 month visit differs from other health visits?**

*Self-Efficacy*

9. **Do you feel confident in supporting your baby’s growth, manage illnesses, or prepare for milestones like weaning?**
   *Probes:* *Attribution to health visits or other factors supporting parents*

Domain: Outer Setting

*Patient Needs & Resources*

10. **How useful are health visits in connecting parents to other resources available to support you and your child?**
    *Probes:* *3-4 month health visit specifically*

**Closing Questions**

- Is there anything you would change?
- **Do you feel that the service should continue offering the 3-4 month review to all parents?**

Probes: Why, why not

- **Is there anything else you’d like to share about your experience or ideas for improvement?**

**Wrap-Up (2–3 minutes)**

**Thank the parent for their time and insights:**
*"We truly appreciate you sharing your experience. This will help us improve the service to better support families."*

- *Ask how best to get £20 gift voucher to carer.*
- *Would you like us to keep you updated on the programme’s development?*

**Public Health Intervention Responsive Studies Teams (PHIRST):**

PHIRST Light Evaluation of the Rotherham Healthy Child Programme.

**CFIR-based Interview Schedule for staff and stakeholders**

**Introduction:**

Thank you for taking the time to speak with me today. We’re looking at the Rotherham Healthy Child Programme, especially the 3–4 month health review. As part of this work, we’re talking to Health Visitors and other staff to understand how the programme is working and how it can be improved. This interview is part of a larger study to gather feedback from different people involved.

The interview will take up to one hour. Your feedback is really important and will help us improve the programme.

In this discussion, we’ll talk about:

- Your thoughts on the programme and the timing of the health reviews.
- The benefits and challenges of the 3–4 month health review for staff, parents, and children.
- How the review supports children’s development and health, and how it fits into the rest of the programme.
- Any ideas on how the programme is working and what could be improved.

Your input will help us make the programme better for everyone involved.

**(For interviewer: Questions in bold, with probes beneath)**

**SSI Questions:**

1. **Can you please introduce yourself, including your current role?**

*(Probe: Can you tell me about your career background/experience)*

*(Probe: this may include – areas you work within Rotherham, length in current post, career background and experience?)*

*(Probe: Can you describe the type of contact you have with parents/carers (if direct facing role)?)*

*Innovation Characteristics*

*Core Intervention Components*

2. **In your opinion, what do you think are the core elements of the Rotherham Healthy Child Programme?**
   - Probe: What do you see as the main purpose of the reviews?
   - Probe: Which elements do you see as the most important for supporting families?
   - Probe: Is it clear to parents and staff why the review has been offered universally?
   - Probe: Have your thoughts changed over time?

*Knowledge & Beliefs about the Innovation*

*Relative Advantage*

3. **All children in Rotherham can currently have a health review at 3–4 months of age. What are your thoughts on having this review for:**
   - Children: What are the benefits and challenges?
   - Parents/carers: What are the benefits and challenges?
   - Health workers/staff: What are the perceived or experienced benefits and challenges?

*Evidence Strength & Quality*

4. **For commissioning staff: How was the decision made to introduce a universal 3-4 month review?**
   - Probe: What were the commissioning decisions? Were there public health priorities that affected the decision?
5. **Do you think the 3–4 month review impacts developmental outcomes, such as Ages and Stages Questionnaire-3 scores at 12 months?**
   - Probe: Can you provide examples of how it does or doesn’t impact these scores?
   - Probe: How long do you think any benefits last? Are there drop-offs?
   - How is the 3-4 month review similar/different to other reviews/check-in’s
   - Probe: What would happen without the 3–4 month review?
   - Probe for managers/commissioners: (Attribution): Contribution and impact of other organisations on ASQ-3 scores?

*Adaptability*

5. **How well does the 3–4 month review fit into most families’ routines?**
   - Probe: Are there any challenges in scheduling or accessibility?
   - Any different/underserved family routines (e.g. cultural differences)
   - Probe: Could the review be delivered differently to better meet family needs?

*Complexity*

6. **How straightforward is the process of delivering the 3–4 month review?**
   - Probe: Are there any tools, such as the ASQ-3, that are difficult to use?
   - Probe: Are there aspects of the review that could be simplified?

*Outer Setting*

*Patient Needs & Resources*

7. **Does the 3–4 month review address the needs of families?**
   - Probe: Are there specific areas where it supports parents or children well?
   - Probe: Are there unmet needs the review could focus on?
8. **Are there challenges that prevent families from engaging with the review?**
   - Probe: Are there barriers like location, timing, or awareness of the review? Cultural or religious barriers?
   - Probe: How can we improve engagement with parents/carers, particularly hard to reach/excluded populations?
9. **Has adding the 3-4 month review impacted you and your role?**
   - Probe: If so, what impact?
   - Probe: Has the review changed your satisfaction with your role?

*Follow-Up Referrals*

9. **Does the 3–4 month review lead to follow-ups or referrals for parents or children? If yes, how beneficial are these referrals?**
   - Probe: Can you share examples of referrals related to breastfeeding, immunisation, or other services?
   - Probe: Are there ways to improve the follow-up process?
   - Probe managers/commissioners:
   - Key agencies/partners and roles
   - Thoughts on referral/partnership work arrangements –	policies/protocols
   - Communications and systems – referral processes	benefits/challenges
   - Service ‘fit’/duplication (added value of 3-4 month review)

*Individual Characteristics*

*Self-Efficacy*

11. **Does the 3–4 month review help parents feel more confident about supporting their child’s development?**
    - Probe: Can you provide examples of how it does or doesn’t build confidence?
    - Probe: Are there areas where parents remain unsure?
12. **For clinical staff: Do you feel confident to deliver the 3-4 month review?**
    - Probe: Why/why not? What would help you feel more confident/ what has helped you feel confident?

*Inner Setting*

*Implementation Climate*

12. **How well does the 3–4 month review integrate into the wider Healthy Child Programme?**
    - Probe: Are there overlaps or gaps with other reviews?
13. **For clinical staff: Do you feel supported by the 0-19 service to deliver this additional visit?**
    - Probe: Why/why not? What would help you feel supported?

Process

*Engaging*

13. **What skills and competencies are essential for ROLE [health visitors/nursery nurses] delivering the 3–4 month review?**
    - Probe: Are there gaps in training or professional development?
    - Probe: Are there specific skills that make the review successful?
14. **How can we improve parent/carer engagement in the 3–4 month review?**
    - Probe: Are there better ways to communicate the importance of the review?
    - How can we adapt to reach families who are harder to engage?

***Reflecting & Evaluating***

15. **What are the main points or priorities the evaluation team should focus on?**
    - Probe: Are there specific outcomes or methods that should be captured?
    - Probe: How can findings be effectively shared with stakeholders?

**Closing Questions**

16. **Are there any suggestions you’d like to make about the longer-term sustainability of the 3–4 month review?**
    - Probe: What changes or investments are needed to maintain the programme?
17. **Is there anything else you’d like to share about the programme or the review?**
    - Probe: Are there any key points we haven’t covered today?

**Wrap-Up**

**Closing Statement:**
*"Thank you for your time and insights today. Your feedback is crucial to helping us improve the Rotherham Healthy Child Programme. If you have any additional thoughts after today, please don’t hesitate to reach out."*
