# Supplementary material for: Evaluation of a universal 3-4 month Healthy Child Programme visit in a local area of England: a mixed methods study
Source: Public Health Pract (Oxf). 2026 Jul 31;12:100837. doi: 10.1016/j.puhip.2026.100837 (PMC13473960; doi:10.1016/j.puhip.2026.100837)
Supplement: Supplementary file 2 [file mmc2.docx]

## **Supplementary Material B**

## **Table: Pre- and post-intervention demographics for those with recorded 9-12 month ASQ score**

|  | | **Pre-intervention ASQ** | | **Post- intervention ASQ** | |
| --- | --- | --- | --- | --- | --- |
| **Characteristic** | **Category** | **n** | **%** | **n** | **%** |
| **Mother age band** | <20 | 20 | 5.2% | 39 | 3.4% |
|  | 20–24 | 58 | 15.2% | 154 | 13.3% |
|  | 25–29 | 118 | 31.0% | 299 | 25.8% |
|  | 30–34 | 112 | 29.4% | 379 | 32.6% |
|  | ≥35 | 63 | 16.5% | 256 | 22.0% |
|  | Not known | 10 | 2.6% | 34 | 2.9% |
| **Child age at 9-12 visit** | 7-8 | 1 | 0.3% | 2 | 0.2% |
|  | 9-10 | 292 | 76.6% | 872 | 75.1% |
|  | 11-12 | 82 | 21.5% | 269 | 23.2% |
|  | 13-14 | 4 | 1.0% | 16 | 1.4% |
|  | 15+ | 1 | 0.3% | 1 | 0.1% |
|  | Not known | 1 | 0.3% | 1 | 0.1% |
| **Ethnicity** | Asian | 44 | 11.5% | 114 | 9.8% |
|  | Black | 21 | 5.5% | 68 | 5.9% |
|  | Mixed | 25 | 6.6% | 48 | 4.1% |
|  | Other | 4 | 1.0% | 11 | 0.9% |
|  | White | 282 | 74.0% | 892 | 76.8% |
|  | Not known | 5 | 1.3% | 28 | 2.4% |
| **Gender** | F | 193 | 50.7% | 555 | 47.8% |
|  | M | 188 | 49.3% | 606 | 52.2% |
| **Gravida** | Primigravida | 157 | 41.2% | 461 | 39.7% |
|  | Multigravida | 143 | 37.5% | 474 | 40.8% |
|  | Not known | 81 | 21.3% | 226 | 19.5% |
| **IMD decile** | 1 | 100 | 26.2% | 269 | 23.2% |
|  | 2 | 57 | 15.0% | 166 | 14.3% |
|  | 3 | 43 | 11.3% | 165 | 14.2% |
|  | 4 | 29 | 7.6% | 112 | 9.6% |
|  | 5 | 27 | 7.1% | 62 | 5.3% |
|  | 6 | 26 | 6.8% | 76 | 6.5% |
|  | 7 | 31 | 8.1% | 129 | 11.1% |
|  | 8 | 15 | 3.9% | 52 | 4.5% |
|  | 9 | 15 | 3.9% | 57 | 4.9% |
|  | 10 | 0 | 0% | 0 | 0% |
|  | Not known | 38 | 10.0% | 73 | 6.3% |
