# Supplementary material for: Evaluation of a universal 3-4 month Healthy Child Programme visit in a local area of England: a mixed methods study
Source: Public Health Pract (Oxf). 2026 Jul 31;12:100837. doi: 10.1016/j.puhip.2026.100837 (PMC13473960; doi:10.1016/j.puhip.2026.100837)
Supplement: Supplementary file 3 [file mmc3.docx]

**Supplementary material C**

**Comparison of numerical ASQ-3 scores at 9-12 month visit, pre- vs post-intervention**

| **ASQ-3 domain** | **Adjusted mean difference (95% CI)** | **p-value** |
| --- | --- | --- |
| Communication | 0.504 (-0.155 to 1.162) | 0.13 |
| Gross motor | 0.928 (-0.04 to 1.897) | 0.06 |
| Fine motor | -0.065 (-0.508 to 0.378) | 0.77 |
| Problem solving | 0.678 (0.190 – 1.166) | <0.01 |
| Personal social | 0.602 (0.023 – 1.182) | 0.04 |

*Multiple linear regression adjusted for IMD quintile, age of mother, gravida, ethnicity, birth month.*
