# Supplementary material for: Evaluation of a universal 3-4 month Healthy Child Programme visit in a local area of England: a mixed methods study
Source: Public Health Pract (Oxf). 2026 Jul 31;12:100837. doi: 10.1016/j.puhip.2026.100837 (PMC13473960; doi:10.1016/j.puhip.2026.100837)
Supplement: Supplementary file 4 [file mmc4.docx]

## **Supplementary Material D**

**SD Table 1: Demographic characteristics of parents participating in the qualitative study by group**

| Demographic characteristics | Individual interviews n = 15 | Mosque focus group n = 17 |
| --- | --- | --- |
| Gender  Female  Male | 15 100)  0(0) | 6(86)  1(14) |
| Ethnicity  White British  Asian or Asian British | 13 (87)  2(13) | 6(86)  1(14) |
| Relationship status  Married  In a relationship  In a civil partnership  Single | 6(40)  7(47)  1(7)  1(7) | 3(43)  2(29)  1(14)  1(14) |
| Sexuality  Heterosexual  Gay/lesbian  Bisexual | 14(93)  0(0.00)  1(7) | 6(86)  1(14)  0(0) |
| Number of children  1 child  2 children  3 children  >3 children | 7(47)  7(47)  1(7)  0(0) | 0(0)  3(43)  2(29)  2(29) |
| Took up the 3-4 month visit?  Yes  No | 15(100)  0(0) | 5(71)  2(29) |

**SD Table 2: Qualitative themes and quotes by CFIR domain and construct**

Innovation: Relative Advantage

| Theme | Primary Factors | Quotes |
| --- | --- | --- |
| Added utility of 3-4 month visit | - Parents: Found it helpful to have contact between the mandated visits, especially for new parents. - Staff: Enjoyed delivering the visit and felt it was a helpful check-in for parents. Felt visit allows early identification and intervention, reducing likelihood of future referrals. | “I think it is a great support to have in there. As I said, with my first I didn't get that and I was a new parent, never done it before, so it was very much a ‘you're in the dark and figure it out’. So I do think it's a great step to have in the children's development steps and check-ups.” (Participant 009)  “The thing with the three to four month review is when we didn’t have it previously, a child universally wouldn’t be seen from six to eight weeks to nine to twelve months, so you're potentially going into a family that’s not been given all the information that is relevant for that current stage in life so they might have missed it. So you potentially are looking at more referrals if they’ve not had this contact as a health professional”. (Nursery nurse) |

Innovation: Innovation Design

| Theme | Primary Factors | Quotes |
| --- | --- | --- |
| Visit setting | - Parents: In-person home visit perceived to be more convenient than medical centre or hub. Home setting may lead to parent openness - *Minor theme:* Home visit may lead to scrutiny and discomfort for some parents - *Minor theme:* Some parents would be happy with group setting or online, but minority | “But I like the face-to-face. I like that they come to your house. And I think if you are in a position that you are struggling, you're definitely more in a position to open up in your own house, like that one to one.” (Participant 006)  “When you do it in your own home, some people might feel a bit uncomfortable with that. And particularly around the checking the sleep space and making sure, I guess the pressure on the parents or the house potentially to look tidy and everything, for a health professional to come into your house. They might feel that they're being overly scrutinized.” (Participant 014) |
| Length of visit | - Parents: Longer visit, allowed more to be covered and parents to feel heard - Staff: Longer visit length gives greater time for discussion and facilitates information retention | “It was a really good review. It lasted longer. It was more informative compared to the eight week review, where that was just purely a health check, to see how I'm doing, see how baby's doing.” (Participant 005)  “It’s like a crucial time to speak with parents because that information can be reiterated, what’s not always taken in from the new births or the six to eight weeks.“ (Nursery nurse) |
| Visit topics | - Parents: Perception that visit repeats topics covered at 6-8 week visit, aids information retention and gave greater focus on developmental milestones - Staff: Nursery nurses chosen as primary deliverers due to skills in developmental screening and identification, allows greater focus on development | “I think the three to four month review is just like I think it's just it repeats a lot of the - isn't the one before that the six to eight week review? Yeah. I think it just repeats a lot of that, apart from new milestones as they're growing, I think a lot of it is repetitive.” (Participant 001)  “One of the reasons why we focused on the Nursery Nurses, so there's a couple of different groups that do the three-to-four-month visit… a lot of their focus is on child development… they're already very skilled on that screening and identification. And you know, doing the ASQ. So yeah, the decision was to kind of use Nursery Nurses for that because then they can kind of track that journey throughout.” (Local authority stakeholder) |
| Timing of visit | - Parents: Felt the visit should have been slightly later (approx. 4-5 months) to align with weaning, particularly for premature babies - *Minor theme:* Some felt it was the right time and understood why it was delivered at 3-4 months - Staff: Commissioning decision to offer 3-4 month window based on evidence about key developmental milestones, ability to counter misinformation, and identify issues early - Staff: Flexibility in timing of review important to ensure equitable access | “For me, I thought it was a bit - because the information that's coming is about six months plus, so it kind of felt not the right time” (FG1 Participant)  “I can see a visit is necessary before six months just purely to give weaning advice because I've heard some parents [start at 4 months] and it's like I'm sure you're not supposed to do it til six months”. (FG1 Participant)  “I think it's about the right time because we know that parents go back to work, it's a key point in weaning and a key point in - and weaning's so like important in terms of communication, speech and language. There's so much linked around that weaning period and feeding and children start to become mobile so it's a really key point in development that you can really do lots of education, prevention. I'm not sure if the parents would prefer it later, I don't know that information but in terms of getting in at the right time to help improve outcomes for children I think it's right.” (Local authority stakeholder)  “I guess because we’re going to them at a time that suits them, you know it doesn’t have to be on the three months exactly, you know it’s quite a wide time span. But when we’ve been looking at kind of the numbers who are not taking it up there isn’t anything specific around underserved or ethnic minorities not taking it up” (Local authority stakeholder) |
| Variability in delivery | - Parents: Perceived inconsistency in elements of the visit delivered and varying advice between nursery nurses, leading to confusion and mistrust. | “One thing that has come up like when I've been talking to other mums that have had their three to four month review is that there seems to be some inconsistency in the checks that are done, so some of us have had length, height measured and some of us didn't.” (Participant 011)  “They like tell you different things, so I don't know what it's like for everyone else, but it just confused me. So they told me to use purees and things like that and then they said not to use purees, to just give her basically what I eat because saliva and her gums she'll be able to eat it, so then I were like well do I give purees or do I give her solid food?”  (Participant 001) |

Innovation: Innovation complexity

| Theme | Key factors | Quotes |
| --- | --- | --- |
| Visit burden | - Parents: Perceived visit to be low burden, found completion of ASQ straight forward although it wasn’t consistently reviewed - Staff: Visit perceived to be higher burden given staffing and resource levels | “I mean, I felt like the form was fine, I weren't informed how to fill it in because obviously on the back it says between one and four it means this and whatever, but just by reading the form I kind of understood what to do”. (Participant 004)  “I think for us, we're busy, very busy, nonstop, especially we've got lots of other commitments with the CDC (Child Development Centre) referrals and things like that. So, it is a lovely, you know, we like to do that visit, but it has sort of put extra pressure on, for my team anyway, on capacity and things like that. I don't know if other areas have found that, it's been, you know, we've juggled it, but it's been hard going. And without another staff member it would have been quite tricky.” (Nursery Nurse) |

Innovation: Innovation Adaptability

| Theme | Key factor | Quotes |
| --- | --- | --- |
| Information adaptability | - Parents: Advice perceived to be culturally adaptable; tailoring of advice is a priority but some required greater detail and better adaptation to child’s needs. - Staff: Flexibility in delivery of information in person-centred approach; priority is to tailor advice to the needs of parents and children. | “When it came to circumcision, I weren't sure and she told me what to do, how to do it and I were really a bit shocked thinking ‘great she gave me the advice for circumcision and that’, yeah, so she got all that support and advice for circumcision.” (FG2 Participant)  “I've had a case before where I've gone into the home and we spoke about floor time, tummy time and things, but the room is just not appropriate for the baby to be on the floor. So it's giving the advice to the parents ‘oh we could move this and rearrange’ and then you've maybe done a follow up call or something and the parents have actually seen the benefits of changing things around, especially if it's a first time mum, it gives them a lot of reassurance and advice if they've not had it before.” (Nursery Nurse) |

Individuals: Capability

| Theme | Key factor | Quotes |
| --- | --- | --- |
| Improved parental knowledge | - Parents: Advice during visit improved parents’ knowledge and confidence of key developmental milestones, including weaning, and was particularly helpful for first time parents. - Parents: ASQ is a helpful tool for understanding child’s developmental, but has the potential to cause worry - Parents: Parental knowledge and experience impacted fit of the visit; having children recently or having related skills made visit less relevant | “Very positive and very informative. I gained a lot of confidence from the review in terms of how to care for my baby, next steps with weaning and teething when it eventually comes”.  (Participant 005)  “It [ASQ] were quite good but again, like she said, you can see your baby's progress, what they're supposed to be doing and stuff like that. I was worried that she wasn't doing certain things or whatever but, yeah, it's completely normal”. (FG1 Participant)  “With my daughter it were good, I needed the support, but with each time now… so my daughter's only just turned two so it's still quite fresh having a baby, do you know what I mean, it's not like I don't know what I'm doing.” (Participant 003) |
| Provides reassurance | - Parents: Visit provides reassurance about child’s development and parenting behaviours - Minor themes: Reassurance from health visitor counters incorrect advice, but information that challenges parent’s behaviours needs to be delivered in the right way. | “I guess it just helps, it's a bit of a reminder, isn't it, that ‘oh we'll have this milestone coming up next and then that milestone and whatever’, but yeah, I think, yeah, just reassures you that you're doing things right or that you know what to expect, what's coming up.” (Participant 010)  “So I've got a lot of my family, like my grandma, aunties that just put me down saying ‘your milk's not good enough for him, he's not growing’. So they bullied me for ages and they did put me down, but my husband was quite supportive and the health visitors obviously were weighing him and telling me I'm doing fine and stuff. So I think if it weren't for them then I probably would have gone on bottle now”. (FG2 Participant). |

Individuals: Need

| Theme | Key factor | Quotes |
| --- | --- | --- |
| Maternal wellbeing | - Parents: Assessment of mother’s wellbeing is an important topic for the visit; some felt this was covered in detail whilst others felt it was not screened thoroughly - Staff: Maternal mental health is a key topic for the visit, alongside child’s mental health/psychological development. Recognition that additional training may be needed to support nursery nurses to screen children’s mental health. | “I think it really can't be underestimated, the importance around the mental health discussion for parents. I think for me it felt like sometimes that was a little bit glossed over or not really given the opportunity to talk about that enough. I'm really open with how I was feeling and I was quite open to say “Look, I'm struggling here.” But I can imagine if someone wasn't as comfortable to say that, if it wasn't proactively mentioned or discussed, it might go under the radar.” (Participant 014)  “I'd like to see a big focus on mental health improvement and mental health for both the child and the parents. And I think probably if I was to say as well, I think infant mental health is probably an area where some practitioners could/would benefit on a more enhanced upskilling, you know, some CPD on a little bit more about observing a child and how you observe a child at 3 to 4 months and understand how its mental health is developing, because there are very specific ways you can do that.” (Local authority stakeholder) |
| Assessing needs | - Staff: Parent and child needs assessment is key to visit, but need to balance formal assessment with person-centred approach to working with family. | “It's really difficult with ASQs because obviously every child does develop differently and the ASQ doesn't take into account if a child - like for example today I've had a child with cerebral palsy and obviously on the ASQ are scoring low - and it's that reassurance that you've got to give to parents that well actually you probably would expect a child with that need to not be reaching these milestones because she's got a physical disability. So the ASQ is for everyone, is sent to everyone, but it doesn't take into account obviously health conditions as well which, again, it can be worrying, can't it, for parents.” (Nursery nurse) |

Individuals: Innovation deliverers

| Theme | Key factor | Quotes |
| --- | --- | --- |
| Relational connection between staff and parents | - Parents: Position connection with staff put parents at ease; linked to length of visit which allowed for time to ask questions - Parents: Staff perceived to have high level of professionalism which set positive expectations for future care - Parents: Perceived judgment from staff damaged relationship with parents | “I definitely felt heard. She answered all my questions, gave me references if I needed to follow anything up with mental health or with weaning or anything. She definitely listened. Didn't feel rushed at all. She were really lovely”. (Participant 006)  “I think all my visits have been by people that are really, really friendly and professional and I think that really helps. I think she obviously was very informative but, yeah, I think that's one of the most important things I suppose just making someone feel at ease, you know, making you aware that they're there for you if you need anything, so maybe you don't need anything at that point, but you might do in the future, so it's good to know that you've got their contact details and things if you do have any concerns in the future that you can obviously pick back up with them and maybe make another appointment and things, so I think that's important to know”. (Participant 002)  “She'd judge you without…um…like you could never have done right in her eyes and you've got to follow the book's way in being a mum, being a parent, a baby should do this at this time, do this at that time. I felt she were a very judgy person, only here for half an hour, but proper looked down her nose at you, sort of thing”. (Participant 003) |

Inner setting: Communications

| Theme | Key factor | Quotes |
| --- | --- | --- |
| Signposting | - Parents: Found the signposting element of the visit very helpful, specifically for weaning and parenting support groups - Staff: Viewed signposting as an important tool in supporting healthy child development and preventing later difficulties | “Actually the lady who came out were really good because she'd signposted to some - not services as such in terms of weigh-ins or whatever - but like actually some of the other stuff that they're doing in Rotherham, so like the baby massage stuff and the play groups and weaning courses.” (Participant 012)  “It's just more informal signposting just to family hubs and talking about things that are happening at different places within the area where they can go and socialise with other mums, different things that might be happening in local parks. We don't tend to really get many referrals at three to four month unless it is maybe something to do with mum's mental health or something like that as such. But it just kind of prevents it then before nine to twelve if they can get out into local area.” (Nursery nurse) |
| Greater clarity of communication | - Parents: Felt that greater clarity around the aims of the visit were needed, including the topics covered and longer length of the visit | “I remember when it came up to the review, I wasn't too sure what it was going to involve, other than the weight which we knew about anyway. And the text that we got sent with the date just said 3-4 month review, didn't give us any information as to why or include a link or anything which might have been helpful. I think what would have been helpful as well would have been to give us an idea of how long it would take. I think she ended up being here about an hour or just over an hour which was longer than what we were expecting.” (Participant 014) |
| Mandatory visits | - Parents: Perceived visit to be mandatory, receiving a letter with visit date. One parent tried to opt out but was unable. - Staff: Belief that 3-4 month visit requires active consent process | “I said to her ‘I don't want them to come round because it's just about feeding, make sure he sleeps safe’, which obviously I already knew, but they wouldn't let me pass up on it.” (Participant 003)  “I'm pretty confident that right from the word go, so from the new birth visit onwards, we always say in health visiting that these visits are mandated and we need to offer them to you but it's your choice. We come to you with your consent and that is clear from the go. And if anybody declines, there's no questions asked. So, I'm pretty confident that right from the very beginning of a relationship with the health visiting team they know that although they're mandated and we have to offer, they don't have to take them.” (Local authority stakeholder) |
| Between-service and within-service communication | - Staff: Communication between services and to parents has been enhanced to reduce barriers for staff and parents. Skilful communication is seen as key element of nursery nurse role. | “So child development is key, that's what their expertise is.  They're highly trained Nursery Nurses so building child development, communication skills I would say is really important, communicating with obviously the parents, especially at the 3 to 4 month stage. Observational skills, so observing and assessing what they're seeing.  Things like motivational interviewing, those sorts of things and solution focused approaches in terms of discussing and identifying goals for families.  Obviously, there will be challenging conversations potentially.” (Commissioner stakeholder) |

Inner setting: Information technology infrastructure

| Theme | Key factor | Quotes |
| --- | --- | --- |
| Ease of contact and communication | - Parents: Found it easy to contact the service and receive communications, particularly text messages. Easy to rearrange appointments. | “I asked at the time for like a morning appointment because my daughter's at nursery and she only does mornings and, again, I didn't want to put anybody out picking her up, so I did ask them and she said to me `oh don't worry, I'll put you down', just like pencilled it in provisionally, ‘as like the first appointment, I think we get there for about half past nine', and then, or quarter past nine, something like that, she says `so I'll pencil you in for that' and when the appointment came through it was perfect, it was at that time (FG1 Participant) |

Inner setting: Work infrastructure

| Theme | Key factor | Quotes |
| --- | --- | --- |
| Staff consistency | - Parents: Consistency in staff delivering visits helps to build a relationship with staff member, encouraging openness to ask questions are share. - Staff: Supported notion that staff consistency is the best approach, but visit setup (i.e. with nursery nurses not health visitors) and staff resourcing mean this does not always happen. | “I think I just didn't see the same person enough. And I think because the visits are very few and far between, I wouldn't say that I built very good rapport, I mean, I can get on with anyone, I can have a discussion and conversation with anyone and I feel that I'm confident enough that I can ask questions that I need to for the benefit of my children to anyone, but I think those who aren't maybe as confident or, you know, it might be difficult if they're not seeing the same person.” (FG2 Participant)  “They'll often see one health visitor at new birth and then somebody else at six to eight weeks, so then from three to four months it is better that they see the same team, isn't it, because they'll be forever seeing different faces and going through things over and over.” (Nursery Nurse) |
| Timing window | - Parents: Found the full day window difficult to plan around and felt it disrupted their day. | Definitely a smaller timeslot, because I think an eight til six, nine til six, eight til six, whatever it is, that's too long of a slot to wait in, especially if you've got more than one kid.” (Participant 003) |

Outer setting: External Pressures

| Theme | Key factor | Quotes |
| --- | --- | --- |
| Macropolitical context | - Staff: Central government decisions around family hub funding, have led to uncertainty and impacted planning within the service. | “At the moment there are 75 local authorities that are directly supported to develop their family hubs. That's roughly half of all the local authority that there are. So it could be that they will extend the programme to be effectively across the whole country, which might then mean that we get a smaller slice of the cake.” (Local authority stakeholder) |

Implementation process: Reflecting and evaluating

| Theme | Key factor | Quotes |
| --- | --- | --- |
| Local data collection | - Staff: Visit was based on formal and informal evidence-base. Local data collection is needed to review impact of visit and make commissioning decisions. | “I think as long as we can evidence that, you know, it's having an impact locally, then I think it's kind of really I guess for myself, and (staff member) to kind of yeah, make the financial case and I think that's always a difficult one. And I think, yeah, what, you know, it is balancing up that we do have kind of I guess like say a set budget and you know, is that three to four month review I guess more important than anything else we do.” (Local authority stakeholder) |
